# Coupling molecular data and experimental crosses sheds light about species delineation: a case study with the genus ***Ciona***

**DOI:** 10.1038/s41598-018-19811-2

**Published:** 2018-01-24

**Authors:** Marine Malfant, Sébastien Darras, Frédérique Viard

**Affiliations:** 1 Sorbonne Universite, CNRS - UMR 7144 ‘AD2M’ - Station Biologique, Roscoff, 29680 France; 2 Sorbonne Université, CNRS, Biologie Intégrative des Organismes Marins (BIOM), Observatoire Océanologique, F-66650 Banyuls/Mer, France

## Abstract

Molecular studies sometimes reveal evolutionary divergence within accepted species. Such findings can initiate taxonomic revision, as exemplified in the formerly recognized species *Ciona intestinalis*. While an increasing number of studies have examined the ecology, reproductive barriers and genetics of *C*. *intestinalis* and *C*. *robusta*, there are still much uncertainties regarding other species of this genus. Using experimental crosses and mitochondrial data, we investigated the evolutionary relationships among four native and introduced *Ciona spp*., found in sympatry in the Mediterranean Sea or English Channel. Outcome of 62 bi-parental reciprocal crosses between *C*. *intestinalis*, *C*. *robusta, C*. *roulei* and *C*. *edwardsi* showed that *C*. *edwardsi* is reproductively isolated from the other taxa, which is in agreement with its distinct location in the phylogenetic tree. Conversely, hybrids are easily obtained in both direction when crossing *C*. *intestinalis* and *C*. *roulei*, reinforcing the hypothesis of two genetically differentiated lineages but likely being from a same species. Altogether, this study sheds light on the evolutionary relationship in this complex genus. It also calls for further investigation notably based on genome-wide investigation to better describe the evolutionary history within the genus *Ciona*, a challenging task in a changing world where biological introductions are shuffling species distribution.

## Introduction

Species can be defined as a group of lineages evolving separately from all others lineages, beginning with a speciation event and ending with its extinction or new speciation events^1,2^. Several species concept have been proposed, each of them relying on specific criteria such as reproductive isolation, biogeographic patterns, molecular divergence or phenotypes (including morphology)^1^. These various concepts reflect that evolutionary and ecological processes impact the divergence among groups of individuals with various effects and speed (e.g. accumulation of genetic changes, niche segregation, increased reproductive isolation). During the period of accumulation of differences, the ‘gray zone’ as coined by De Queiroz^1^, different groups of individuals undergoing a speciation process may be distinguished by some criteria, for instance deep genetic divergence, but not necessarily with other, for instance they may lack complete reproductive isolation. Speciation is a dynamic process which results from step-by-step accumulation of these differences of various nature. All the species concepts, and associated criteria, mentioned above are thus of interest, and jointly used in integrative taxonomy studies^2–5^.

In marine taxa, the joint use of biological species and phylogenetic concepts are particularly important regarding the difficulty to use other, for instance those based on biogeography and morphology. The natural distribution range of many marine species has indeed been strongly modified by human activities through biological introduction processes, leading Carlton^6^ to define cryptogenic species, i.e. species for which native *versus* non-native status is undetermined. Biological introductions not only shuffle species distribution and break biogeographic boundaries but also promote hybridization between previously isolated taxa, and thus changes in species evolutionary dynamics, (^7^; e.g. in *Mytilus* spp.,^8^). Molecular tools have revealed numerous cryptic species, i.e. species morphologically indistinguishable, across all biogeographical regions^9^. In the marine realm, the proportion of undiscovered molecular cryptic species had been estimated to vary from 11 to 43% of species described across 49 *infra-ordo*, with the highest proportion found in taxa with few diagnostic external morphological characters^10^. Cryptic taxa are often revealed by population genetic studies: unexpected genetic divergence and low gene flow within accepted species have often been a first step towards an investigation of the species status^4^. Whenever possible, taxonomic revision also benefits from experimental crosses testing for reproductive isolation (in support of the biological species concept). The biological species concept, as compared to the other ones, interestingly does not rely on *a priori* definition of biological units: reproductive isolation observed when using experimental crosses is a sufficient proof that two lineages have evolved separately.

The species complex *Ciona intestinalis* exemplifies the need to merge diverse approaches to achieve species delineation. In the early 2000’s, several molecular studies^11–15^ reveal that the formerly accepted species *C*. *intestinalis*, first described by Linnaeus^16^ was made of four cryptic distinct lineages that were named type A, B, C and D by Zhan, *et al*.^13^. Further studies focused on *C*. *intestinalis* type A and *C*. *intestinalis* type B. The two taxa have been introduced in different part of the world, in particular type A, putatively native to Asia, displayed a worldwide distribution^17^. Genome-wide studies showed a clear genetic distinctiveness between the two taxa, even in their range of sympatry in Europe, with a divergence time about 4 Myr^17–23^. Based on these molecular evidences and on morphological features^19,20^, type B and type A were finally reclassified as *C*. *intestinalis sensu* Millar and *C*. *robusta* (described by Hoshino and Tokioka^24^), respectively^25^. It is noteworthy that despite their clear distinctiveness, the two species lack reproductive isolation under laboratory conditions^26,27^ and hybridize (without marked introgression) in the wild in their area of sympatry in NE Atlantic^26^.

Interestingly, two accepted species, *Ciona roulei*^28^ and *C*. *edwardsi*^29^, are living in sympatry with the introduced and newly recognized species, *C*. *robusta*, in the Mediterranean Sea. *Ciona roulei* and *C*. *edwardsi* are putatively native to this region where they were first described. However, little is known about the relationships (i.e. evolutionary divergence, reproductive isolation) among the three taxa, and with *C*. *intestinalis*. For instance, Nydam and Harrison^11,18^ made detailed investigation of the phylogenetic relationships within the *Ciona* genus but did not include *C*. *edwardsi* in their studies.

Also it is not always straightforward to use previous published studies regarding these four species, because of the unknown date of introduction of *C*. *robusta* in the NE Atlantic and Mediterranean Sea^17^ and the recent taxonomic revision: until 2015, only *C*. *intestinalis* was accepted and thus reported in publications. For example, Lambert, *et al*.^30^ studied the reproductive isolation between *C*. *edwardsi, C*. *roulei* and “*C*. *intestinalis*” (Table 1). However, the latest is more likely to be *C*. *robusta* with the new nomenclature, as the individuals were collected in the Mediterranean Sea and along the Californian coasts, where only *C*. *robusta* has been reported so far. Based on this assumption, it can be hypothesized that Lambert, *et al*.^30^ showed that *C*. *edwardsi* is reproductively isolated from *C*. *robusta* whereas *C*. *robusta* and *C*. *roulei* can reproduce with each other, with asymmetric success. This has nevertheless to be confirmed in light of the new nomenclature, and the reproductive isolation between *C*. *roulei* and the newly reclassified *C*. *intestinalis* has still to be investigated. Reproductive success between taxa is a major criteria for determining if different taxa could potentially be merged or split into different species. This is particularly important to examine for *C*. *roulei* and *C*. *intestinalis*. *C*. *roulei* is an accepted species described for a long time but molecular phylogenetic studies failed to recognize this taxa as a distinct species from *C*. *intestinalis*^11,18^. It is likely that the two species are actually two lineages of the same species. If this is true, absence of reproductive isolation between *C*. *roulei* and *C*. *robusta* should be observed as for crosses between *C*. *intestinalis* and *C*. *robusta* with the same asymmetry (Table 1).

**Table 1:** Main outcomes of laboratory crosses obtained in previous studies, with the four *Ciona* species studied.

Original name	Putative name	Sampling area	Main outcome (with original species name)	Reference
*Ciona savignyi* *Ciona intestinalis*	*Ciona savignyi* *Ciona robusta*	California (USA)	Full reproductive isolation	Lambert, *et al*.^30^
*Ciona intestinalis* *Ciona roulei* *Ciona edwardsi*	*Ciona robusta* *Ciona roulei* *Ciona edwardsi*	Banyuls-sur-Mer (France)	Partial reproductive isolation (asymmetry) between *C*. *roulei* and *C*. *intestinalis*	
			Full reprodutive isolation of *C*. *edwardsi* with *C*.*roulei* and *C*. *intestinalis*	
*Ciona intestinalis* *Ciona intestinalis*	*Ciona intestinalis* *Ciona robusta*	Celtic Seas (Scotland, UK) NW Pacific (Kyoto, Japan)	Partial reproductive isolation between specimens of Japanese vs. Scottish origin (fertilization rate: 2.3%-70.4%)	Suzuki, *et al*.^14^
*Ciona intestinalis* type A *Ciona intestinalis* type B	*Ciona robusta* *Ciona intestinalis*	Plymouth (UK) and Naples (Italy) Plymouth (UK)	*C*. *intestinalis* type A and B isolated	Caputi, *et al*.^15^
			Poor success of allopatric crosses with *C*. *intestinalis* type A	
*Ciona intestinalis* type A *Ciona intestinalis* type B	*Ciona robusta* *Ciona intestinalis*	English Channel (Plymouth, UK)	No reproductive isolation (no asymmetry)	Sato, *et al*.^22^
*Ciona intestinalis* type A *Ciona intestinalis* type B	*Ciona robusta* *Ciona intestinalis*	English Channel (Plymouth, UK)	no reproductive isolation, hybrids F1 fertile, back-crosses possible	Sato, *et al*.^27^
*Ciona intestinalis* type A *Ciona intestinalis* type B	*Ciona robusta* *Ciona intestinalis*	English Channel (Moulin Blanc, France) English Channel (Aber Wrac’h, France)	Partially isolated (asymmetry)	Bouchemousse, *et al*.^26^
*Ciona robusta* *Ciona intestinalis*	*Ciona robusta* *Ciona intestinalis*	English Channel (Moulin Blanc, France) English Channel (Aber Wrac’h, France)	No isolation using *C*. *intestinalis* as maternal lineage	Malfant *et al*.^53^

“Original name” corresponds to the species name used in the paper whereas “Putative name” is the species name that we propose to be the most likely following the taxonomic revision and nomenclature accepted from September 2015.

Finally, reproductive incompatibilities can also vary with the geographic origin (and the associated eco-evolutionary history). For instance, Caputi, *et al*.^15^ failed to produce juveniles from homospecific crosses of *C*. *robusta* collected in Plymouth (UK, English Channel) and Naples (Italy, Mediterranean Sea) (Table 1), raising the question of the variability in reproductive compatibility between populations of *C*. *robusta* and other species of the same genus. The extent of reproductive isolation between *C*. *robusta* collected in the Mediterranean Sea *versus* NE Atlantic with *C*. *roulei* and *C*. *edwardsi* remains to be ascertained.

To resolve the uncertainties listed above and clarify the evolutionary relationships between European *Ciona* spp., we carried out 1) a phylogenetic study using mitochondrial data with specimens collected over the same area; our study provides the first molecular data for *C*. *edwardsi* as compared to other *Ciona* species (native or introduced) found in Europe and 2) crossing experiments to examine in details the extent of reproductive isolation between populations or/and species within the *C*. *intestinalis*/*C*. *robusta* species complex and other *Ciona* spp. found in sympatry. We examined success rate at different stages of the life-cycle, focusing on early-stages, which are the most sensitive for marine invertebrates^31^, using reciprocal bi-parental crosses. We then discussed the relationships between reproductive isolation and mitochondrial genetic divergence.

## Results

### Mitochondrial Molecular inferences

**Figure 1:**
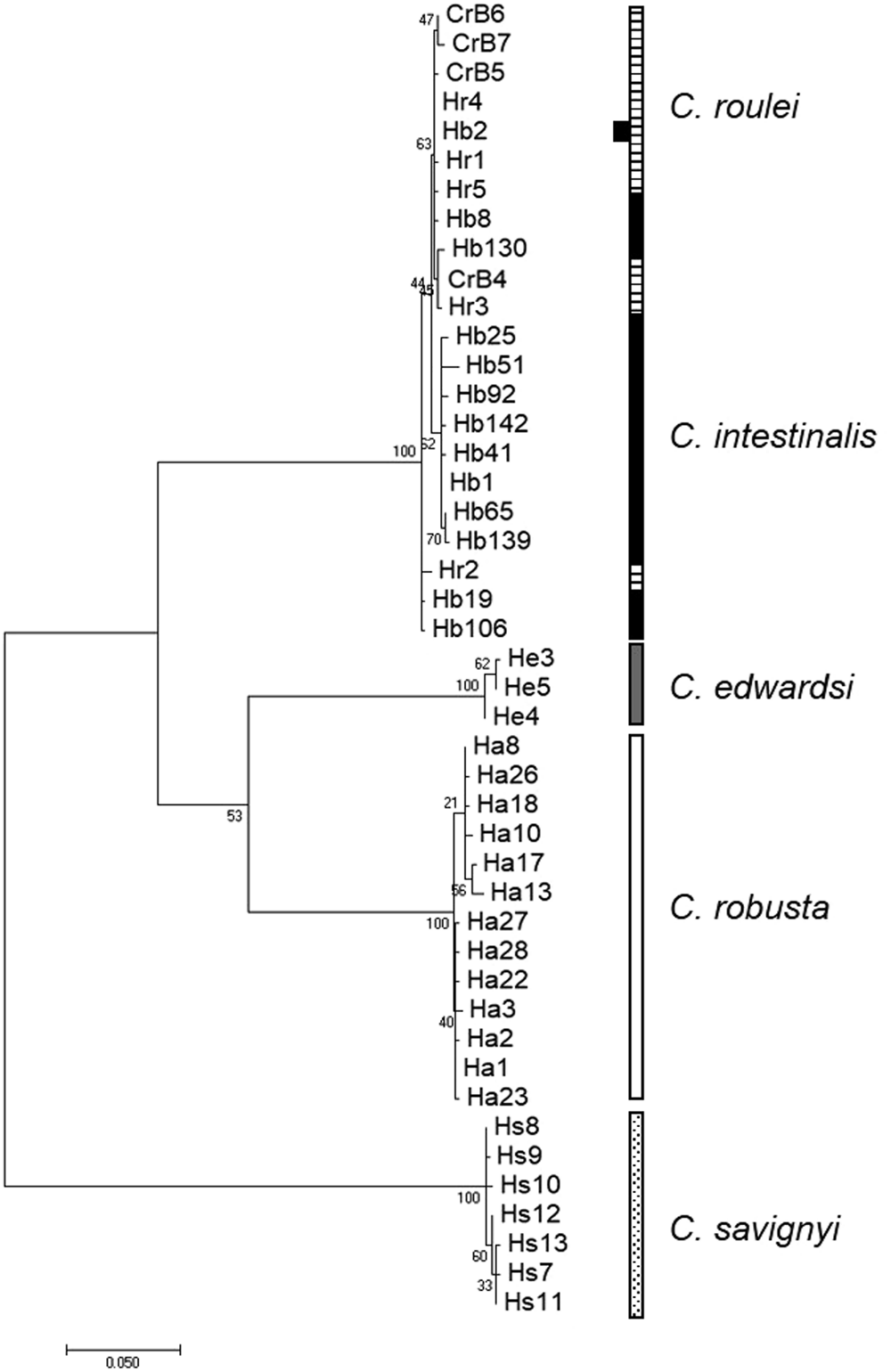
Molecular phylogenetic tree constructed using the Maximum Likelihood method based on the T92 model with 67% of sites evolutionarily invariant. The tree was built with 42 unique sequences obtained (1) from 33 individuals used in this study for experimental crosses and (2) 15 additional haplotype^17^ (see Material & Methods). The tree is drawn to scale, with branch lengths measured in the number of substitutions per site.

Altogether, we obtained 37 mitochondrial sequences over 717 base pairs, including the 33 individuals used in crossing experiments and the four additional *C*. *edwardsi* individuals, which led to the definition of 27 different haplotypes for the four targeted species (see details in Table S2). The six and nine individuals of *C*. *edwardsi* and *C*. *roulei*, sampled for this study were described by three and seven haplotypes, respectively. For *C*. *roulei*, six of the haplotypes found in this study were new as compared to those described by Nydam and Harrison^11^ and one (CrB4) was found in the two studies. Two and one haplotypes were new for *C*. *robusta* and *C*. *intestinalis*, respectively, as compared to the haplotypes described in Bouchemousse, *et al*.^17^. The phylogenetic tree was finally build-up with 46 unique sequences (Table S1). The phylogenetic tree displayed in Fig. 1 shows four well-supported (99–100%) monophyletic groups made of all sequences associated to (1) *C*. *savignyi* (used an outgroup and rooting the tree), (2) *C*. *robusta*, without differences between specimens collected in the English Channel or in the Mediterranean Sea, (3) *C*. *edwardsi*, and (4) *C*. *roulei* and *C*.*intestinalis*, the sequences of the two latter mixed with each other in the tree, with one haplotype (Hb2) shared by both *C*. *roulei* and *C*. *intestinalis* specimens (Fig. 1). It is noteworthy that the relative clustering of *C*. *edwardsi* as compared to *C*. *robusta* and *C*. *intestinalis* is poorly supported by bootstrap values. All the results above were supported using a distance method (Neighbor-joining; 1000 bootstraps) showing the same topology (Fig. S1) and by simple pairwise comparison based on the number of substitutions between sequences (Table S3).

### Reproductive success. Early stages (fertilization to hatching)

For the crosses made with *C*. *edwardsi*, no fertilization could be observed before two hours. Egg cleavage took more time to be observed in this species as compared to the other ones (not shown). Because of this delay, the first measure of reproductive success (i.e. fertilization success) in *C*. *edwardsi* could not be compared with the other types of crosses. Note however that this result does not question the quality of the sperm and eggs of the two individuals used as hatching rates and survival was high (see below).

**Figure 2:**
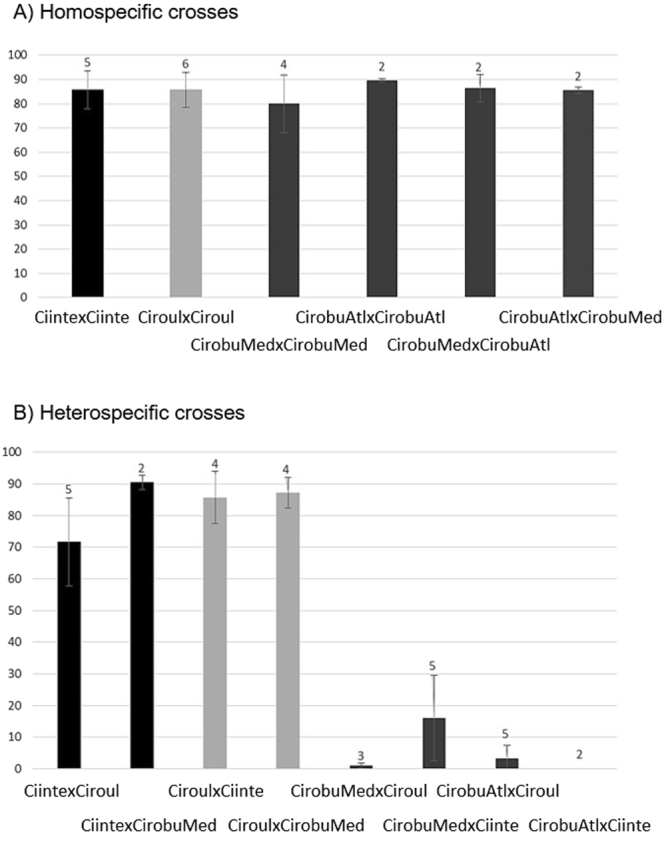
Mean and standard deviation of fertilization rate (%) obtained with (**A**) homospecific crosses with different colors per species. (**B**) Heterospecific crosses (labelled with maternal lineage first and corresponding color). The number of bi-parental crosses per category is indicated above each bar. Abbreviations: CirobuMed, *C*. *robusta* from Mediterranean Sea; CirobuAtl, *C*. *robusta* from English Channel; Ciinte, *C*. *intestinalis*; Ciroul, *C*. *roulei*.

Excluding *C*. *edwardsi* all homospecific crosses worked well, as expected, with more than 80% of fertilization success (Fig. 2A). The geographic origin of *C*. *robusta* also did not affect the fertilization success within this species (see ‘CirobuMed x CirobuAtl’ in Fig. 2A).

Interestingly, many of the heterospecific crosses also showed very good fertilization success. In particular, crosses between *C*. *intestinalis* and *C*. *roulei* led on average to 78% of fertilization success, as compared to 85% of success for each of homospecific crosses for these two species (Fig. 2B). The same holds when using oocytes of one of these two species with sperm of *C*. *robusta* (on average, 88.4% of fertilization success). The reciprocal crosses (i.e. *C*. *robusta* used as a mother) were however not successful (7% on average with either *C*. *intestinalis* or *C*. *roulei*). The geographical origin seems however to play a role (Fig. 2B): the mean fertilization rate of oocytes of *C*. *robusta* collected in Mediterranean Sea by sperm of *C*. *intestinalis* was 15% (n = 5 crosses, and one cross reached 50%), whereas fertilization success was null when fertilizing oocytes of *C*. *robusta* collected in the English Channel with sperm of *C*. *intestinalis*. Such an effect was not so clearly observed with the sperm of *C*. *roulei* although a slight reverse asymmetry was observed (3.2% and 1.0% for fertilization of eggs of *C*. *robusta* collected in the English Channel and the Mediterranean Sea, respectively).

**Figure 3:**
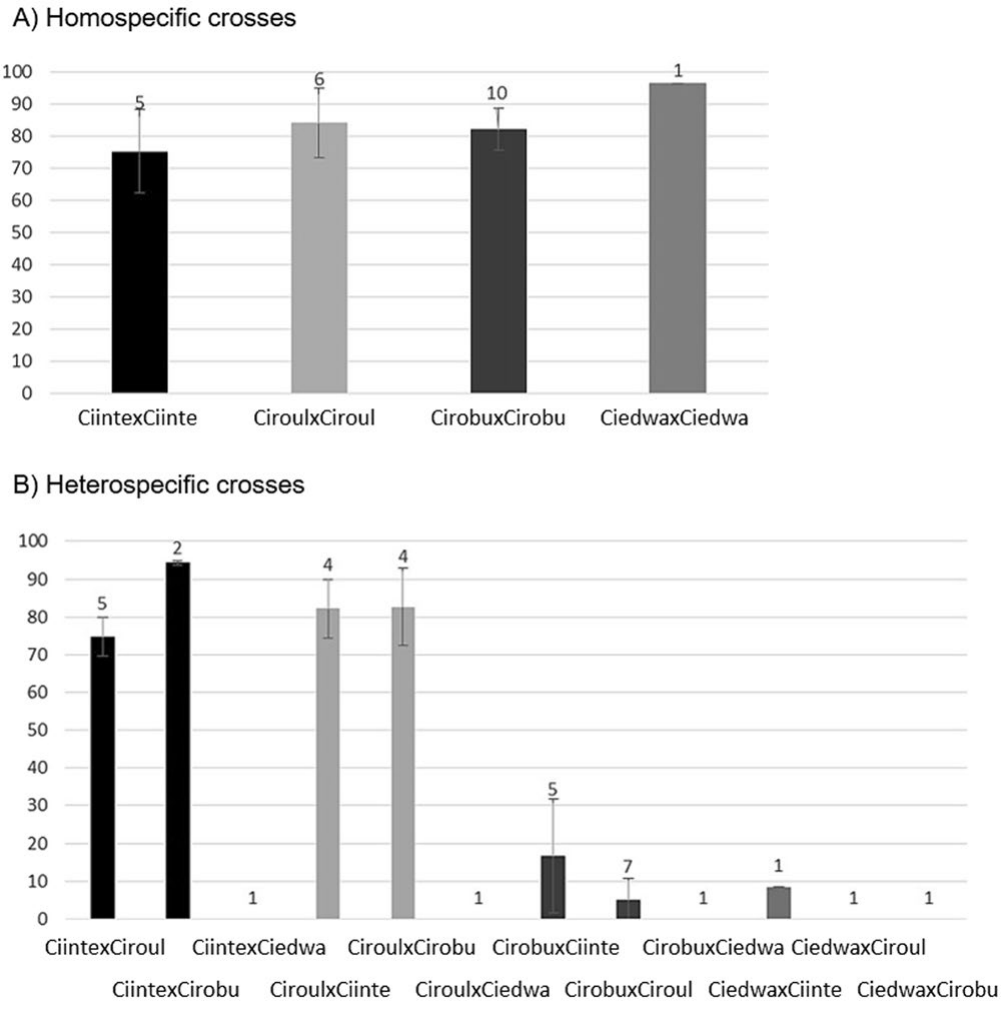
Mean and standard deviation of hatching rate (%) obtained with (**A**) homospecific crosses. (**B**) Heterospecific crosses (labelled with maternal lineage first). The number of bi-parental crosses per category is indicated above each bar. Abbreviations: Cirobu, *C*. *robusta* Ciinte, *C*. *intestinalis*; Ciroul, *C*. *roulei*; Ciedwa, *C*. *edwardsi*.

Hatching rates were similar for all homospecific crosses, including for *C*. *edwardsi* (Fig. 3A), with variations mostly observed within categories (i.e. putative parental effects). Heterospecific crosses showed contrasted results across categories (Fig. 3B): very high hatching rates (>75%) were observed for crosses made with eggs of either *C*. *intestinalis* and *C*. *roulei* with sperm of *C*. *robusta* as well as between *C*. *intestinalis* and *C*. *roulei* whatever the direction of the crosses. Conversely, poor hatching rates were observed in crosses involving eggs of *C*. *robusta* (16.6% ± 15.1% with *C*. *intestinalis* and 5% ± 5.7% with *C*. *roulei*). And no hatching was observed when *C*. *edwardsi* was involved except for one cross made with oocytes of *C*. *edwardsi* and sperm of *C*. *intestinalis* but with low success (8.5% of hatching).

### Survival and growth rate

**Figure 4:**
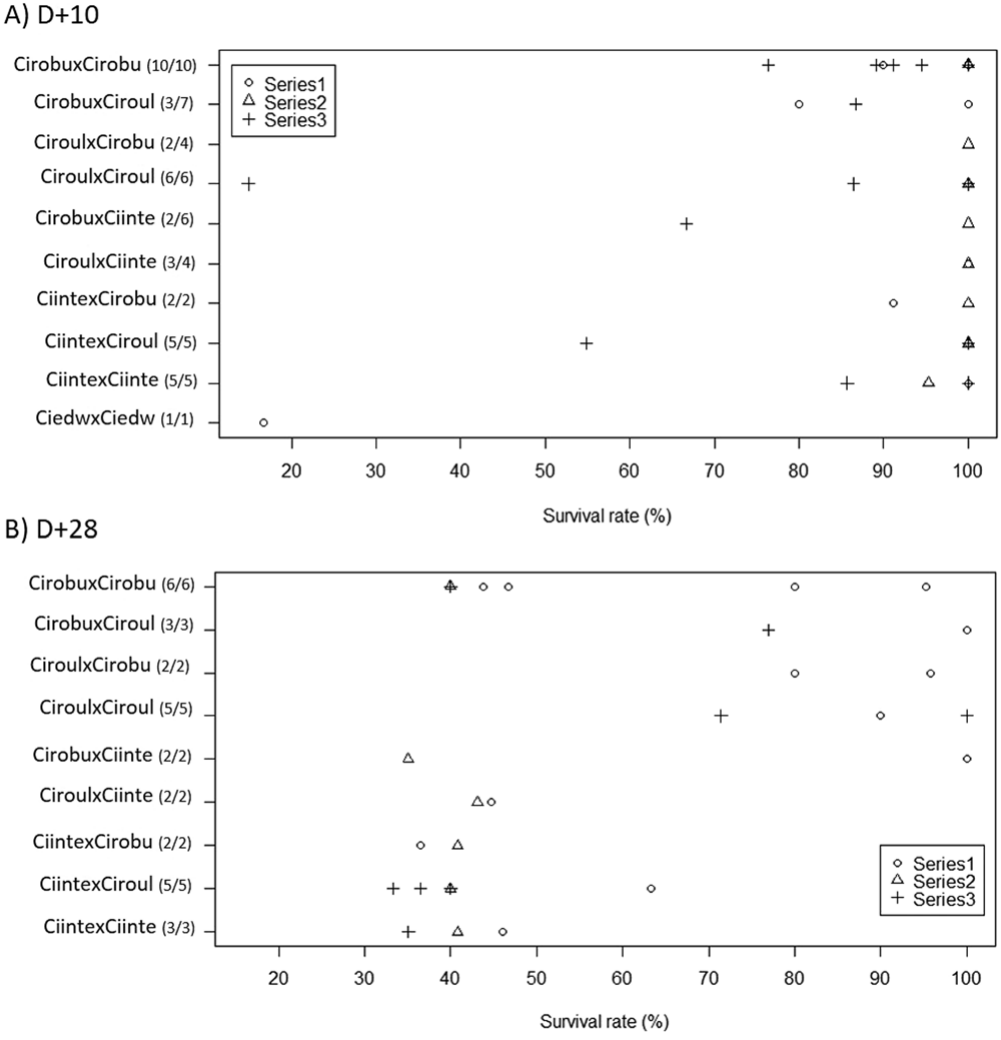
Survival rate per bi-parental crosses at a) D + 10 with survival estimated by comparison with the number of larvae settled at D + 3 and b) D + 28 with survival compared with the number of juveniles alive at D + 10. The biparental crosses are labelled with maternal lineage first; numbers in bracket indicate the number of crosses with live organisms as compared to the total number of crosses surveyed per category over the period. Abbreviations: Ciinte, *C*. *intestinalis*; Ciroul, *C*. *roulei*; Cirobu, *C*. *robusta*; Ciedwa, *C*. *edwardsi*.

For subsequent development, the geographic origin of *C*. *robusta* was not distinguished as results were identical (data not shown). Survival was first examined at D + 10 by comparing the number of juveniles with the number of post-larvae settled at D + 3. At D + 10, the three series were composed of 12, 10 and 17 crosses respectively, with variable categories of crosses within each. A non-parametric Kruskal-Walis test showed significant differences among series (p = 0.024) that may be due to change in food (see Material and Methods). Results are thus presented in Fig. 4 distinguishing both crosses and series. Several crosses were lost (i.e. all post-settled larvae died) between D + 3 and D + 10 in categories involving *C*. *robusta* as a mother and *C*. *roulei* as a father (Fig. 4A). For the other crosses, survival rates was very high for homospecific crosses involving C. *intestinalis* or *C*. *robusta* (90% on average; Fig. 4.A), whatever the series. For *C*. *roulei* one showed high mortality (15% of survival rate) but the three others (from two series) displayed high survival rate (86%). The same hold with heterospecific crosses which showed high survival rates at D + 10, often at level similar to homospecific crosses (Fig. 4A). At the end of the experiment, at D + 28, none of the crosses kept after D + 10 were lost (Fig. 4B) but survival rates were variable across crosses, the lowest values (ca. 30%) being observed for F1-*C*. *intestinalis* and hybrids made with eggs of *C*. *intestinalis* (Ciinte x Ciroul, Ciinte x Cirobu in Fig. 4B).

**Figure 5:**
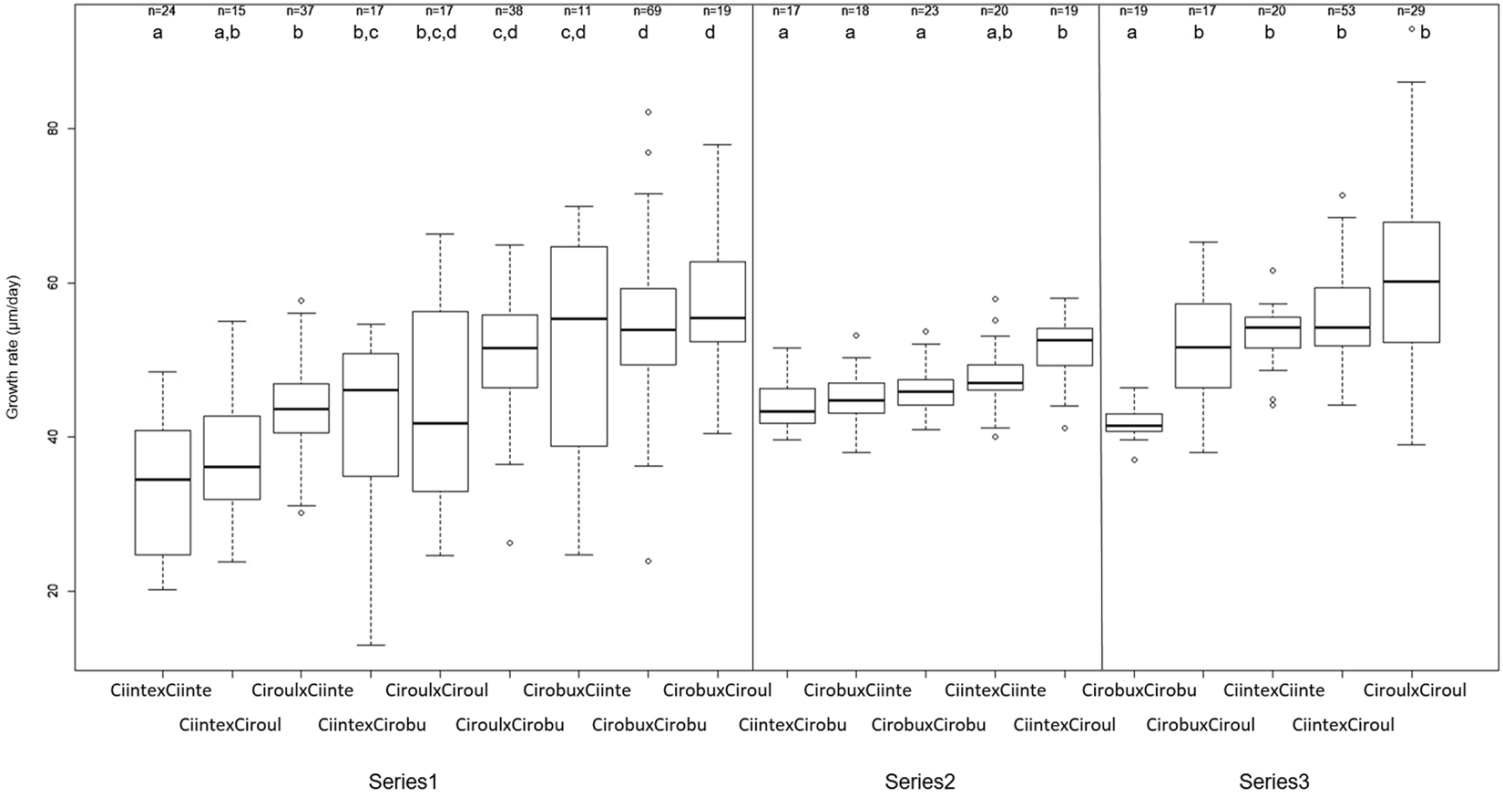
Boxplot showing the first quartile, the median, the third quartile, lower and upper values (coef = 1.5) and extreme values (dots) of growth rate (µm/day) of 28 day-old juveniles (number of juveniles measured is indicated at the top). Results are displayed separately for the three series (see Material and Methods). Within each series, letter on the top of each boxplot indicates different groups distinguished at 5% level by the Ryan-Einot and Gabriel-Welsch test. The biparental crosses are labelled with maternal lineage first. Abbreviations: Ciinte, *C*. *intestinalis*; Ciroul, *C*. *roulei*; Cirobu, *C*. *robusta*.

Growth rates, shown in Fig. 5, were calculated at the end of the experiment (D + 28) using all juveniles alive in each category. Each series was however distinguished for the reasons explained above. An ANOVA confirmed that series as well as the interaction between series and categories have significant effects (p < 0.001 for both). For each of the three series, juvenile’s growth rates were significantly different among categories (Kruskal-Wallis, p-value < 10^−5^ each) but with variable ranking among categories according to series, suggesting either parental effects or/and food influence (series effect). Nevertheless, interestingly, all series show that the growth rates of either F1-*C*. *intestinalis* or F1-*C*. *roulei* were not significantly different from the growth rates of the F1-hybrids between these two species. In series 2, these hybrids even show the highest growth rate.

## Discussion

This study aimed to investigate the evolutionary relationships between four species of the genus *Ciona* living in sympatry in NE Atlantic, namely *C*. *roulei, C*. *intestinalis, C*. *robusta* and *C*. *edwardsi*, by using and comparing the outcome of a mitochondrial phylogenetic tree and the extent of reproductive isolation among these taxa.

Experimental reciprocal bi-parental crosses and subsequent monitoring of survival and growth during 28 days first showed that homospecific crosses developed well, as expected, including when different geographic origins were used. For instance, individuals from two spatially disjoint populations of *C*. *robusta* have been used in crosses: some were sampled in the English Channel and some in the Mediterranean Sea. We did not see any evidence in support of variations in gamete compatibility between populations, conversely to what has been observed by Caputi, *et al*.^15^ who used individuals from Plymouth (English Channel) and Naples (Mediterranean Sea). The low success of crosses made with individuals from Plymouth in the study by Caputi, *et al*.^15^ is more likely due to stressed individuals (long-distance transport or poor acclimatization to local conditions) as already discussed by Sato, *et al*.^27^.

The first main outcome of our experimental study is that heterospecific crosses often showed results similar to homospecific crosses, except when involving *C*. *edwardsi*. Success was particularly high when considering crosses between *C*. *intestinalis* and *C*. *roulei*. Our results show that the two taxa behave like two non-reproductively isolated lineages: they can hybridize with high rate of success in both directions and display survival and growth rates similar to those observed in homospecific crosses of the two taxa. Concerning growth rates, no significant statistical differences were observed with the non-parametric test used. Regarding the low power of such tests, this is noteworthy that F1-*C*. *roulei* showed a higher growth rate than F1-C. *intestinalis*. This may be due to our raising condition (e.g. local filtered seawater) which could be more favorable to the local Mediterranean specimens (*C*. *roulei*) as compared to the Atlantic ones (*C*. *intestinalis*). Similar experiments should be repeated on a much larger number of bi-parental crosses in other places and using different seawaters.

The two taxa also behave similarly regarding the other tested species, in particular *C*. *robusta*. Both *C*. *roulei* and *C*. *intestinalis* hybridize well with *C*. *robusta* but in one direction only (oocytes of *C*. *roulei* and *C*. *intestinalis* could be fertilized by sperm from *C*. *robusta*). Such an asymmetry has been previously described for *C*. *intestinalis* by Bouchemousse, *et al*.^26^ and for *C*. *roulei* by Lambert *et al*.^30^ (note that in the later study*, C*. *robusta* was named *C*. *intestinalis*, following the accepted taxonomy and nomenclature at this time). Bouchemousse, *et al*.^26^ showed that despite (1) high rate of hybridization between *C*. *intestinalis* and *C*. *robusta* in laboratory conditions of and (2) a similar reproductive period in syntopic populations, hybridization between the two taxa is extremely rare, and introgression seems to be absent, in the wild. Extrinsic post-zygotic barriers (i.e. environmental barriers) do not seem neither to be effective against gene flow between the two species in their sympatry area. Hybrids are indeed performing as well as their parental species under various temperature and salinity conditions^53^. It is thus likely that intrinsic barriers, notably due to genomic incompatibilities in second generation hybrids (i.e. Dobzhansky-Muller incompatibilities (DMI)^35–37^), may be at play. Reproductive isolation and DMIs have often been shown to be asymmetrical in plants and animals^56^, in relation with differential selection on male and female functions. The genes involved in such asymmetrical and putative genomic incompatibilities between *C*. *robusta* and *C*. *intestinalis* are unknown. However, it is noteworthy that a self-sterility mechanism has been described in *C*. *robusta* by Harada, *et al*.^54^. These authors have identified two loci, Themis-A and Themis-B, interacting to reject self-fertilization. The Themis-B encodes for two genes, one expressed in eggs (v-ThemisB) and the other in sperm (s-ThemisB). A similar system could be involved in homospecific recognition between the vitelline coat of *C*. *robusta* oocytes and sperm, which do not recognize sperm of another species. Yamada *et al*.^55^ identified 800 proteins in the vitelline coat (VC) of *C*. *robusta*, including v-ThemisA and B. Further researches are needed to investigate the mechanisms of the genetic incompatibilities between *C*. *robusta* and *C*. *intestinalis*. A first step could be to compare VC proteins of *C*. *robusta* with VC proteins of *C*. *intestinalis*. In addition, taking into account our experimental results, it would be particularly interesting to test for the existence of first-generation hybrids between *C*. *robusta* and *C*. *roulei* in their sympatry area, the Western Mediterranean Sea. If results are similar to those observed between *C*. *robusta* and *C*. *roulei* (i.e. absence of introgression in the wild), it would be an ideal situation to look for conservation of the same DMIs across spatial replicates.

Altogether, in light of the biological species concept, the results of experimental crosses clearly support the hypothesis that *C*. *roulei* and *C*. *intestinalis* are actually the same species. The taxonomic hypothesis is strongly supported by the results of the phylogenetic analysis, and thus by a phylogenetic criteria. The mitochondrial tree indeed showed that individuals of the two species are mixed with each other. Lahille^28^ distinguished *C*. *roulei* from *C*. *intestinalis* with morphological criteria such as a longer atrial siphon than the oral one (the reverse being described for *C*. *intestinalis*), a different structure of the branchial wall and a reddish color due to specific pigments in *C*. *roulei*. Surprisingly, Brunetti *et al*.^19^ showed similar branchial structure between *C*. *intestinalis* and *C*. *robusta*. While we did not examine this latter characteristic on our specimens, we measured the relative length of the siphons but did not observe differences between *C*. *roulei* and *C*. *intestinalis*. However, consistent differences in body color were noticed between the *C*. *intestinalis* and *C*. *roulei* specimens, as described by Lahille^28^ (Fig. S2). By contrast, we observed a similar pigmentation of the gonoducts openings (orange pigmentation of the oviduct and absence of pigmentation of the spermiduct), a trait that serves to discriminate *C*. *intestinalis* from *C*. *robusta* (absence of pigmentation of the oviduct and dark red pigmentation of the spermiduct end)^22^. However, color is known to vary across specimens, notably according to the environment, in several species of *Ciona*^19,22^. The contrasted color we observed could thus be due to different regions of sampling for the two taxa (English Channel and Mediterranean Sea for *C*. *intestinalis* and *C*. *roulei*, respectively). Altogether based on Lahille^28^, Brunetti *et al*.^19^ and our study, further investigations by taxonomists are required to determine the robustness of the morphological criteria distinguishing the two taxa. Our phylogenetic tree is also in agreement with results obtained in previous studies that examined at once *C*. *intestinalis* and *C*. *roulei*. In particular, Nydam and Harrison^11,18^ and Zhan *et al*.^13^ clearly showed that *C*. *roulei* and *C*. *intestinalis* do not display reciprocal monophylly, a criteria required to be fitted in support of the phylogenetic species concept for species delineation. Our study confirmed these previous findings, expanding the origin of the individuals studied (including sequences from Bouchemousse, *et al*.^17^). Some marine species described as inhabiting both the Mediterranean Sea and the NE Atlantic were shown to be actually composed of cryptic species or cryptic lineages, such as *Dicentrarchus labrax* also distinct between Atlantic and Mediterranean Sea, when each group are homogenous^38^. However, the reverse situation, similar to the one described with *C*. *roulei* and *C*. *intestinalis* in this study, has also been reported. For instance, the species status of *Pecten maximus* and *P*. *jacobaeus* (L. 1758) is unclear: they have been recognized as two distinct species diverged 5My ago, but recent genetic studies showed low differentiation between the two species based on microsatellites^39^ confirming previous findings based on allozymes^40^ and mitochondrial data^41^. Populations of *P*. *maximus* show more differentiation along the Atlantic coast than with *P*. *jacobeus* in the Western Mediterranean Sea. The Almeria-Oran front has most often been shown to be associated with a strong genetic barrier between populations from Atlantic Ocean and Mediterranean Sea^42,43^ but there are exceptions. Patarnello, *et al*.^42^ hypothesize that populations with no genetic differentiation were separated during the late Pleistocene recolonization of the Mediterranean Sea. To better assess the historical processes that led to the faint genetic distinctiveness of *C*. *roulei* and *C*. *intestinalis*, a population-based study is needed. *C*. *roulei* has, so far, been described and sampled along the Western Mediterranean coast from southern France to northern Spain but the species distribution range is unknown. We thus first need to complement the survey (and sampling) along the Mediterranean coasts down to the Oran-Almeria front and along the NE Atlantic coasts on the other side of this front. Then we need to use nuclear markers to make fine-assessment of the demographic history of the two lineages, using coalescence-based methods to test for alternative divergence scenarios between them. Demographic inferences (e.g. estimate of the time of divergence) could help to understand why populations are now geographically isolated and morphologically distinct but not genetically and reproductively isolated. The two taxa could be at the onset of a speciation process, i.e. in the grey zone as defined by De Queiroz^1^. Roux, *et al*.^44^ shows that pairs of lineages/taxa are in the gray zone when their divergence is between 0.5–2%, which is congruent with our result (based on mitochondrial data) of a divergence of 0.84% between *C*. *roulei* and *C*. *intestinalis*.

Our study also brought new results regarding *C*. *edwardsi*. We showed that *C*. *edwardsi* is strongly divergent from *C*. *intestinalis* and *C*. *robusta* with on average 13.4% and 10.2% of mitochondrial divergence, respectively. Based on these molecular data, and assuming a relationship between evolutionary divergence and reproductive isolation^44,45^, *C*. *edwardsi* is expected to be either partially reproductively isolated with *C*. *robusta*, such a*s* observed between *C*. *robusta* and *C*. *intestinalis*, or completely isolated with *C*. *robusta*, such as observed between *C*. *robusta* and *C*. *savignyi*^46^. Although with a limited number of individuals used (i.e. sperm from one individual and eggs from another one) because of sampling limitations, our experimental crosses clearly showed a complete reproductive isolation between *C*. *edwardsi* and *C*. *intestinalis, C*. *roulei, C*. *robusta*. A few larvae were produced with one cross made with *C*. *intestinalis* but it might be the result of enforced fertilization in petri dishes, as observed sometimes with crosses between eggs of *C*. *robusta* with sperm of *C*. *intestinalis*^26^. In addition, juveniles produced with this cross poorly survived after 10 days post-fertilization (16% of survival rate). To explore the evolutionary relationships between those four species, further studies are nevertheless required, notably to resolve the phylogenetic position of *C*. *edwardsi* as compared to *C*. *robusta* and *C*. *intestinalis*. A phylogenomic approach could be particularly interesting to carry out as they have been shown efficient to better understand relationship in species complex and in taxonomic groups with complex histories of migration and divergence, such as flowering plants (*Pedicularis*), American oak (*Quercus*) and deep-sea octocorals (*Chrysogorgia, Anthomastus-Corallium*)^47–50^. Phylogenies are indeed often complex to resolve when species hybridize along their evolutionary history, which is likely to have occurred among the *Ciona* spp. occurring in sympatry in European waters. Past introgression can indeed explain discordance between nuclear or mitochondrial gene trees as shown on marine *vs* freshwater fish phylogenies^51^; such gene flow can be maintained between highly divergent species as already shown for *C*. *robusta* and *C*. *intestinalis*^23^.

Our results also call for further population genetics investigation of the study species: two haplotypes for *C*. *robusta* and one for *C*. *intestinalis* were new as compared to those published by Bouchemousse, *et al*.^17^. The haplotypes associated to adult specimens of *C*. *robusta* collected in the Mediterranean Sea were not distinguished from the haplotypes of specimens sampled in the English Channel, confirming previous findings by Bouchemousse, *et al*.^17^ of a likely common origin of the populations introduced in the English Channel and along the Western Mediterranean coasts of France. As for *C*. *intestinalis*, we observed a high level of polymorphism for *C*. *roulei* and *C*. *edwardsi*: with only nine and six individuals respectively, no less than five and three haplotypes were found over ca. 700 base pairs, suggesting large effective size of the populations. The challenge here will be to identify the habitats where *C*. *edwardsi* can be sampled as unfortunately little is known about the ecology of this species, although it may be an interesting outgroup for further phylogenetic and genomic studies of the two model species *C*. *intestinalis* and *C*. *robusta*.

## Conclusion

Despite a complex speciation history (divergence with gene flow, e.g. *C*. *robusta* and *C*. *intestinalis*) and background noise due to biological introductions (e.g. *C*. *robusta* introduced in the respective and disjoint native ranges of *C*. *intestinalis* and *C*. *roulei*), our study shows that coupling experimental studies and molecular analyses can help elucidating evolutionary relationships. Our results also give support to the classical assumption of a positive relationship between time of divergence and reproductive isolation: prezygotic barriers are indeed expected to increase with parental divergence even if the degree of divergence needed to the reproductive isolation varies along taxa^45^. In addition, this study confirmed our hypothesis regarding *C*. *roulei* and *C*. *intestinalis*: based on both biological and phylogenetic species concepts and criteria, these two accepted species are more likely to be two isolated populations, genetically poorly distinct, of a same species. At the opposite side of the population-species continuum, *C*. *edwardsi* is clearly a distinct species showing reciprocal monophylly and reproductive isolation with the three other study taxa. To our knowledge, this study was the first study attempting to resolve the phylogenetic relationship of *C*. *edwardsi* with species presumably living in sympatry (or parapatry). Other markers are however required to resolve in details the evolutionary history within this group of taxa, as the use of a single mitochondrial marker, besides some potential limitations due to its nature (maternally inherited, non-recombinant), does not allow fine investigation of historical gene flow and demography. Altogether, our results call for further integrative taxonomy studies and raise questions regarding the extent and barriers of inter-specific gene flow between these species living in sympatry in the wild.

## Material and Methods

### Adult sampling

Details about the samples used are provided in Table S1. Briefly, all adult specimens used in this study were collected by scuba diving in the western Mediterranean Sea (in Banyuls-sur-Mer and Thau Lagoon), except for *C*. *intestinalis* sampled in the English Channel and sent alive to the Banyuls Observatory where the experiments were made. Sampling *C*. *roulei* and *C*. *intestinalis* in different regions where the two taxa are in allopatry help to ensure their identification, in addition to morphological criteria (see discussion and Fig. S2). Specimens of *C*. *robusta* were also collected from the English Channel for sake of comparison between the two European regions (NE Atlantic and Mediterranean Sea) where this species is known to have been introduced. Only a small number of *C*. *roulei* and *C*. *edwardsi* specimens could be sampled (nine and six, respectively) despite repeated surveys, suggesting that the two species are either rare or in unknown (and/or difficult to access to) habitats. *C*. *intestinalis* and *C*. *robusta* were easily distinguished as described by Brunetti *et al*.^19^ *C*. *roulei* and *C*. *edwardsi* individuals were identified using morphological criteria following Lahille^28^ and Copello^52^ respectively. The color of the tunic (orange-reddish for *C*. *roulei* and bright yellow for *C*. *edwardsi*) clearly distinguished the two species. After the use of the adults to produce offspring, a piece of tissue was preserved for further DNA studies (see below).

### Fertilization and juveniles raising

After sampling, individuals were kept in closed tanks with constant light to induce gametogenesis. Not all individuals reached maturity, for instance only two *C*. *edwardsi* specimens out of 6 collected produced enough gametes to be used in bi-parental crosses. A total of 33 individuals were used to make 62 bi-parental homo- (N = 22) and heterospecific (N = 40) crosses using the four species *C*. *roulei, C*. *intestinalis, C*. *robusta* and *C*. *edwardsi*. For logistic constraints, the 62 crosses were split across three series carried out between September 10 and September 25. Table S2 provides details about the number of crosses that were carried out per series and for each categories of crosses.

Experiments were made following protocols provided in Malfant, *et al*.^53^. Briefly, the number of oocytes of one individual was estimated in three drops of 150 µl. Oocytes were then split in equal number into four petri dishes to be fertilized by the sperm of four individuals: one from the same species, to be used as a control, and three belonging to each of the three other tested species, whenever possible. Fertilization was made in a controlled room at 15 °C, with 500 µl of sperm diluted in seawater (5 µl of dry sperm in 2 ml seawater). One hour after fertilization, the fertilization rate was estimated for each cross by counting cleaved eggs. Hatching rates were estimated 24 hours after fertilization by counting larvae. For each bi-parental cross, the larvae were then randomly distributed in petri dishes stored afterwards in a controlled room at 17 °C. Following larval metamorphosis, three days after fertilization, settlement rate was measured. The experiment was pursued for plates with more than 10 settlers at D + 3. Survival rate of juveniles was measured 10 days (D + 10) and 28 days (D + 28) after fertilization. For logistic constraints not all crosses were kept after D + 10 to be surveyed until D + 28 (see results). The experiment was stopped after 28 days with pictures of the individuals taken with a dissecting microscope, for subsequent size analysis. After the opening of the siphons and until the end of the experiment, the juveniles were fed every day with microalgae provided *ad libidum*. Because of technical problems with production of microalgae, food had to be modified over the course of the experiment. The first series of crosses were fed during 10 days with T-*Isochrisis galbana* and *Tetraselmis sueccisa* in proportion 50:50 (produced by local facilities) and then with a mixture of microalgae from Greensea (mainly composed of *Nannocloropsis gaditana)*. The second serie of crosses were fed during 4 days with the microalgae produced locally and then with the Greensea product. The third series of crosses were fed exclusively with Greensea product. Changing food in the course of the experiment obviously did not influence fertilization, settlement and hatching rates but could have had an influence on survival and growth rates measured. Survival and growth rates were thus examined separately for the three series, each included different categories allowing for comparisons (Table S2). Using pictures taken at the end of the experiment (D + 28), the size of each juvenile was measured with Image J and transformed into growth rate (µm/day). Assumptions required for ANOVA not being fulfilled, non-parametric tests were carried out using R^32^ for examining growth rates between F1 categories. Post hoc tests were done using the Steel-Dwass-Critchlow-Fligner test procedure implemented in XLSTAT (Version 2015.4.01, Addinsoft).

### Mitochondrial molecular data analysis

All adults (N = 33) used in this experiment were sequenced on the cytochrome c oxidase subunit I (COI) mitochondrial gene. The COI fragment was amplified using primer sequences and protocols obtained from Nydam and Harrison^11^. We also sequenced four additional individuals of *C*. *edwardsi* collected at the same time but which never reach sexual maturity. Purification of PCR products and sequencing reactions were performed by Eurofins. All PCR products were sequenced in both directions. Sequences obtained were edited using CodonCode Aligner v.4.0.2 (CodonCode Corporation, MA). In addition, we included in the dataset seven sequences (all different) of *C*. *savignyi* that were obtained as part of a side study. This species was used as outgroup of the targeted *Ciona* spp. Finally, we included six and 11 sequences (all different) obtained by Bouchemousse, *et al*.^17^ to be representative of the mitochondrial diversity and known distribution of *C*. *robusta* and *C*. *intestinalis*, respectively, and four sequences of *C*. *roulei* from Nydam and Harrison^11^. All the individuals and/or sequences used in this study are detailed in Table S1 which also provides Genbank accession numbers.

The sequences were aligned using BioEdit v.7.1.981^33^ leading to a 717 base pairs final alignment (all sequences were 737 bp except for the sequences of *C*. *roulei* from Nydam and Harrison^11^ shorter by 20 base pairs at the 5’ start of the sequence). A tree was built with MEGA7^34^ with complete deletion of missing data. Goodness of fit with various evolution model was first tested: based on BIC criteria, the best model explaining the data is the Tamura 3-parameter (T92) model with 67% of the sites evolutionarily invariable. Using AIC criteria, the Best model (ΔAIC > 10) is the General Time Reversible (GTR) model with 63% of the sites evolutionarily invariable (I). The two models were used in subsequent analyses. Results were identical; only results based on T92 are shown. A rooted phylogenetic tree was constructed using a maximum likelihood method with heuristic search (Subtree-Prunning-Regrafting method). Initial tree(s) for the heuristic search were obtained by applying the Neighbor-Joining method to a matrix of pairwise distances estimated using the Maximum Composite Likelihood (MCL) approach. To assess the reliability of the inferred tree, a boostrap test was carried out (1000 bootstraps). Note that similar topology was obtained using other tree construction method, in particular simple distance-based method (i.e. Neighbour-joining).

## Electronic supplementary material

Supplementary Material
