# Supplementary material for: Coupling molecular data and experimental crosses sheds light about species delineation: a case study with the genus Ciona
Source: Sci Rep. 2018 Jan 24;8:1480. doi: 10.1038/s41598-018-19811-2 (PMC5784138; doi:10.1038/s41598-018-19811-2)
Supplement: Supplementary file 1 — Supplementary Material [file 41598_2018_19811_MOESM1_ESM.pdf]

Supplementary Material

Coupling molecular data and experimental crosses sheds light about species delineation: a case study with the genus *Ciona.*

MALFANT Marine, DARRAS Sébastien, VIARD Frédérique

Table S1. Sampling (individuals used in crosses and phylogenetic studies) and mitochondrial haplotypes

For each species, the area and locality where the individuals were collected are indicated. The role (i.e. father, a mother or both) of the individuals used in crosses is shown. Additional data were used for the phylogenetic study as indicated in the table.

Area	Sampling locality	Individual name	Used in crosses	COI haplotype	Genbank accession number	Reference
			Ciona edwardsi			
Mediterranean Sea	Banyuls-sur-Mer	CionaE1	sperm	He3	MG584168	This study
Mediterranean Sea	Banyuls-sur-Mer	CionaE2	eggs	He4	MG584169	This study
Mediterranean Sea	Banyuls-sur-Mer	CionaE3	no	He3	MG584168	This study
Mediterranean Sea	Banyuls-sur-Mer	CionaE4	no	He5	MG584170	This study
Mediterranean Sea	Banyuls-sur-Mer	CionaE5	no	He5	MG584170	This study
Mediterranean Sea	Banyuls-sur-Mer	CionaE6	no	He3	MG584168	This study
			Ciona intestinalis			
NE Atlantic (E. Channel)	Roscoff (Bloscon)	CionaI1	sperm	Hb142	MG584171	This study
NE Atlantic (E. Channel)	Roscoff (Bloscon)	CionaI2	eggs	Hb142	MG584171	This study
NE Atlantic (E. Channel)	Roscoff (Bloscon)	CionaI3	sperm	Hb92	MF479368	This study & Bouchemousse, et al. [17]
NE Atlantic (E. Channel)	Roscoff (Bloscon)	CionaI4	eggs	Hb142	MF479278	This study
NE Atlantic (E. Channel)	Roscoff (Bloscon)	CionaI5	sperm	Hb106	MF479382	This study & Bouchemousse, et al. [17]
NE Atlantic (E. Channel)	Roscoff (Bloscon)	CionaI6	eggs	Hb8	MF479284	This study & Bouchemousse, et al. [17]
NE Atlantic (E. Channel)	Roscoff (Bloscon)	CionaI7	sperm	Hb1	MF479277	This study & Bouchemousse, et al. [17]
NE Atlantic (E. Channel)	Roscoff (Bloscon)	CionaI8	sperm and eggs	Hb25	MF479301	This study & Bouchemousse, et al. [17]
NE Atlantic (E. Channel)	Roscoff (Bloscon)	CionaI9	sperm and eggs	Hb1	MF479277	This study & Bouchemousse, et al. [17]
NE Atlantic (E. Channel)	Perros-Guirrec	Ci-PG-RXIIp-l08	no	Hb130	MF479405	Bouchemousse, et al. [17]
NE Atlantic (E. Channel)	Roscoff (Bloscon)	Ci-BLO-21	no	Hb19	MF479295	Bouchemousse, et al. [17]
NE Atlantic (North Sea)	Gullmar Fjord	Ci-GullF-19	no	Hb51	MF479327	Bouchemousse, et al. [17]
NE Atlantic (North Sea)	Grundsund harbour	Ci-Gru-26	no	Hb65	MF479341	Bouchemousse, et al. [17]
NW Atlantic	Nahant	Ci-NAH-RXIII-05	no	Hb41	MF479317	Bouchemousse, et al. [17]

NW Atlantic	Nahant	Ci-NAH-RXIII-07	no	Hb139	MF479414	Bouchemousse, et al. [17]
			Ciona roulei			
Mediterranean Sea	Banyuls-sur-Mer	Cionar1	sperm	Hr1	MG584174	This study
Mediterranean Sea	Banyuls-sur-Mer	Cionar2	eggs	CrB4	EF209101.1	This study & Nydam and Harrison [11]
Mediterranean Sea	Banyuls-sur-Mer	Cionar3	sperm and eggs	Hr2	MG584175	This study
Mediterranean Sea	Banyuls-sur-Mer	Cionar4	sperm	Hr3	MG584176	This study
Mediterranean Sea	Banyuls-sur-Mer	Cionar5	eggs	CrB4	EF209101.1	This study & Nydam and Harrison [11]
Mediterranean Sea	Banyuls-sur-Mer	Cionar6	sperm	Hr4	MG584177	This study
Mediterranean Sea	Banyuls-sur-Mer	Cionar7	sperm and eggs	Hb2 (1)	MF479278	This study & Bouchemousse, et al. [17]
Mediterranean Sea	Banyuls-sur-Mer	Cionar8	sperm and eggs	Hb2 (1)	MF479278	This study & Bouchemousse, et al. [17]
Mediterranean Sea	Banyuls-sur-Mer	Cionar9	sperm and eggs	Hr5	MG584178	This study
Mediterranean Sea	Banyuls-sur-Mer		no	CrB4	EF209101.1	Nydam and Harrison[11]
Mediterranean Sea	Banyuls-sur-Mer		no	CrB5	EF209102.1	Nydam and Harrison[11]
Mediterranean Sea	Banyuls-sur-Mer		no	CrB6	EF209103.1	Nydam and Harrison[11]
Mediterranean Sea	Banyuls-sur-Mer		no	CrB7	EF209104.1	Nydam and Harrison[11]
			Ciona robusta			
NE Atlantic (E. Channel)	Brest (Moulin Blanc)	CionaRatl1	sperm	Ha1	MF479417	This study & Bouchemousse, et al. [17]
NE Atlantic (E. Channel)	Brest (Moulin Blanc)	CionaRatl2	eggs	Ha1	MF479417	This study & Bouchemousse, et al. [17]
NE Atlantic (E. Channel)	Brest (Moulin Blanc)	CionaRatl3	sperm	Ha1	MF479417	This study & Bouchemousse, et al. [17]
NE Atlantic (E. Channel)	Brest (Moulin Blanc)	CionaRatl4	eggs	Ha1	MF479417	This study & Bouchemousse, et al. [17]
NE Atlantic (E. Channel)	Brest (Moulin Blanc)	CionaRatl5	eggs	Ha1	MF479417	This study & Bouchemousse, et al. [17]
NE Atlantic (E. Channel)	Brest (Moulin Blanc)	CiA-MB-16	no	Ha2	MF479418	Bouchemousse, et al. [17]
NE Atlantic (E. Channel)	Plymouth	Ci-QAB-48	no	Ha3	MF479419	Bouchemousse, et al. [17]
Mediterranean Sea	Thau lagoon (Sète)	CionaRmed1	sperm	Ha27	MG584172	This study
Mediterranean Sea	Thau lagoon (Sète)	CionaRmed2	eggs	Ha1	MF479417	This study & Bouchemousse, et al. [17]
Mediterranean Sea	Thau lagoon (Sète)	CionaRmed3	sperm	Ha1	MF479417	This study & Bouchemousse, et al. [17]
Mediterranean Sea	Thau lagoon (Sète)	CionaRmed4	eggs	Ha3	MF479419	This study & Bouchemousse, et al. [17]
Mediterranean Sea	Thau lagoon (Sète)	CionaRmed5	eggs	Ha1	MF479417	This study & Bouchemousse, et al. [17]
Mediterranean Sea	Thau lagoon (Sète)	CionaRmed6	sperm	Ha1	MF479417	This study & Bouchemousse, et al. [17]

Mediterranean Sea	Thau lagoon (Sète)	CionaRmed7	eggs	Ha1	MF479417	This study & Bouchemousse, et al. [17]
Mediterranean Sea	Thau lagoon (Sète)	CionaRmed8	sperm	Ha28	MG584173	This study
Mediterranean Sea	Thau lagoon (Sète)	Ci-SET-RXIII-10	no	Ha22	MF479438	Bouchemousse, et al. [17]
Mediterranean Sea	Thau lagoon (Sète)	Ci-SET-RXIII-11	no	Ha23	MF479439	Bouchemousse, et al. [17]
Mediterranean Sea	Naples	Ci-NaplRXI-05	no	Ha1	MF479417	Bouchemousse, et al. [17]
NE Pacific	Monterey	Ci-PN2-109	no	Ha26	MF479442	Bouchemousse, et al. [17]
NE Pacific	Monterey	Ci-PN2-116	no	Ha18	MF479434	Bouchemousse, et al. [17]
SE Pacific	Coquimbo	Ci-Herra-1584	no	Ha8	MF479424	Bouchemousse, et al. [17]
SE Pacific	Ganaqueros	Ci-Guana-1679	no	Ha13	MF479429	Bouchemousse, et al. [17]
NW Pacific	Tokyo	Ci-TOK-D-09	no	Ha10	MF479426	Bouchemousse, et al. [17]
NW Pacific	Ichimonji	Ci-ICHI-03	no	Ha17	MF479433	Bouchemousse, et al. [17]
			Ciona savignyi			
NW Pacific	Mutotsu	Cs Mutotsu 1	no	Hs7	MG584179	This study
NW Pacific	Mutotsu	Cs Mutotsu 2	no	Hs8	MG584180	This study
NW Pacific	Mutotsu	Cs Mutotsu 3	no	Hs10	MG584182	This study
NW Pacific	Mutotsu	Cs Mutotsu 4	no	Hs11	MG584183	This study
NW Pacific	Mutotsu	Cs Mutotsu 11	no	Hs9	MG584181	This study
NW Pacific	Takenoura	Cs Takenoura 3	no	Hs12	MG584184	This study
NW Pacific	Takenoura	Cs Takenoura 12	no	Hs13	MG584185	This study

(1) The haplotype Hb2 has been described for *C. intestinalis* by Bouchemousse, Bishop *et al.* 2016 (Hb2 was found in 25 localities from NE and NW Atlantic) as well as for *C. roulei* in this study and a previous study (Bouchemousse, S. & Viard, F., unpublished data) with specimens sampled in 2013 in Banyuls sur Mer.

References:

11 Nydam, M. L. & Harrison, R. G. Genealogical relationships within and among shallow-water *Ciona* species (Ascidiacea). *MAR BIOL* **151**, 1839-1847, doi:10.1007/s00227007-0617-0 (2007).

17 Bouchemousse, S., Bishop, J. D. & Viard, F. Contrasting global genetic patterns in two biologically similar, widespread and invasive *Ciona* species (Tunicata, Ascidiacea). *SCI REP-UK* **6**, 24875, doi:10.1038/srep24875 (2016).

Table S2. Number of bi-parental crosses per categories (i.e. combination of taxa tested).

All crosses that could have been made with the different targeted taxa or geographic origins are listed. Ciinte, Ciroul, Ciroumed, Cirouatl, Ciedwa stand for *Ciona intestinalis*, *C. roulei*, *C. robusta* from the Mediterranean Sea, *C. robusta* from the English Channel and *C. edwardsi*, respectively. Homospecific crosses (used as controls) are highlighted in gray.

The series refer to the three set of bi-parental crosses carried out between Sept 10 and Sept 25, 2016. The three series are distinguished because they did not experienced exactly the same food all along the experiment. This may have an effect on survival and growth rate estimates, which were therefore analyzed separately for the three series. Most of the categories were repeated in the three series.

eggs	sperm	Series 1	Series 2	Series 3	Total
Ciinte	Ciinte	1	1	3	5
Ciinte	Ciroul	1	1	3	5
Ciinte	Cirobumed	1	1	-	2
Ciinte	Ciedwa	1	1	-	2
Ciroul	Ciroul	2	1	3	6
Ciroul	Ciinte	2	2	-	4
Ciroul	Cirobumed	2	2	-	4
Ciroul	Ciedwa	2	2	-	4
Cirobumed	Cirobuatl	1	1	-	2
Cirobumed	Cirobumed	1	1	2	4
Cirobumed	Ciinte	1	1	2	4
Cirobumed	Ciroul	1		2	3
Cirobumed	Ciedwa	1	1	1	3
Cirobuatl	Cirobuatl	1	-	1	2
Cirobuatl	Cirobumed	1	-	1	2
Cirobuatl	Ciinte	-	-	2	2
Cirobuatl	Ciroul	1	1	3	5
Cirobuatl	Ciedwa	-	-	-	0
Ciedwa	Ciedwa		1		1
Ciedwa	Ciinte	-	1	-	1
Ciedwa	Ciroul				0
Ciedwa	Cirobumed		1		1
Ciedwa	Cirobuatl	-	-	-	0
Total		20	19	23	62

Table S3. Number of pairwise differences between the COI sequences obtained for the five study species over 717 base pairs.

	C. edwardsi	*C. savignyi*	*C. robusta*	*C. intestinalis*
C. savignyi	98			
C. robusta	73	97		
C. intestinalis	96	113	83	
C. roulei	96	114	85	6

Fig. S1. Evolutionary relationship between the five species inferred using the Neighbor-Joining method, bootstraps value (%) are next to the branches (1000 bootstraps).

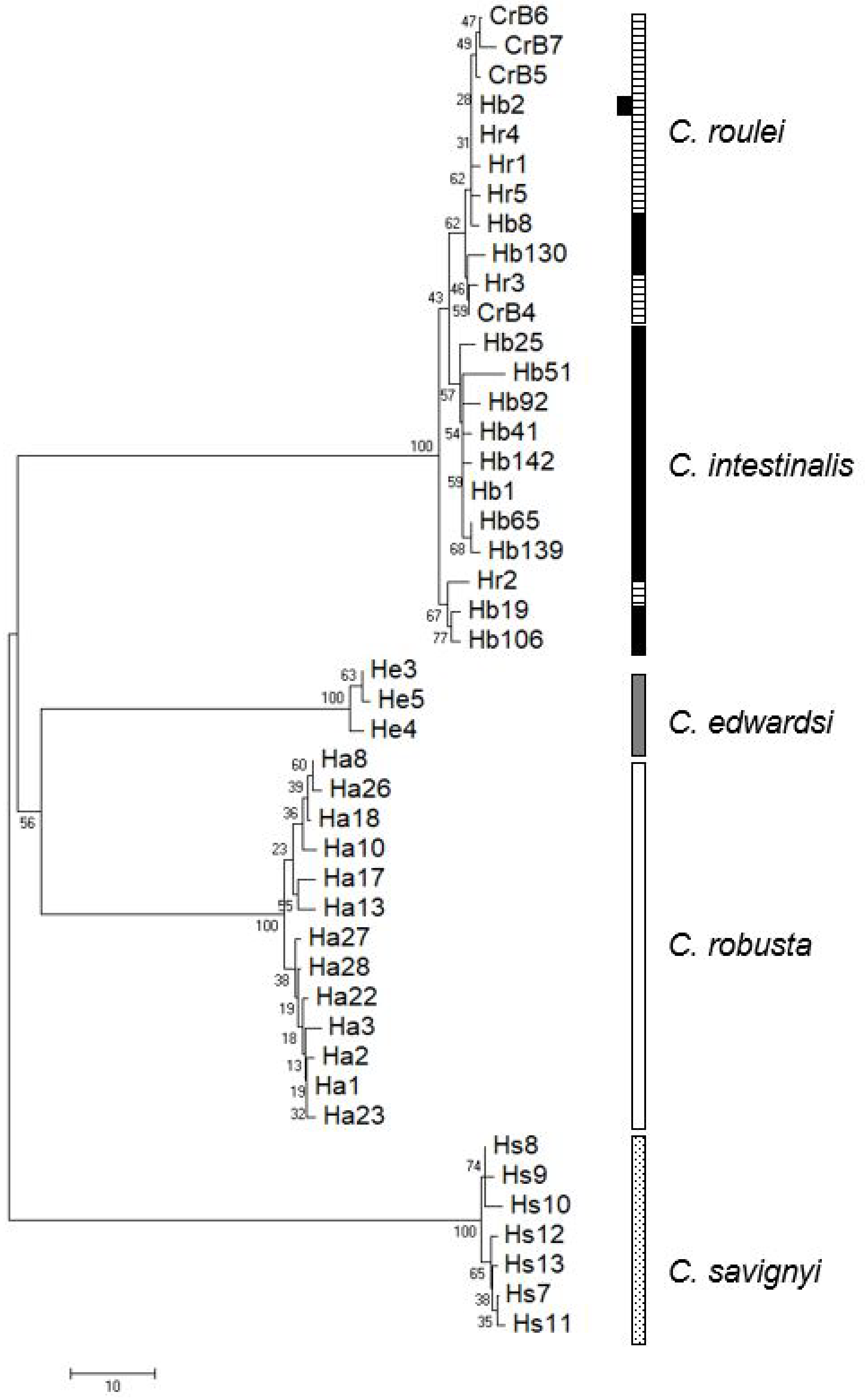

Fig. S2. Pictures of the four study *Ciona* species at different stages of the life cycle.

C. intestinalis (A, E, I and M). *C. roulei* (B, F, J and O). *C. robusta* (C, G, K and N). *C. edwardsi* (D, H, L and P).

(A-D) Live adults; note that the pictures A to D are not at the same scale. The presence of specific tubercular prominences in *C. robusta* is visible around siphons [19]. *C. intestinalis* is translucent [19], *C. roulei* is orange/red pigmented [28] and *C. edwardsi* is bright yellow [52].

(E-H) Close views of the distal/anterior ends of the gonoduct of the fours taxa following dissection; the spermiduct is readily visible by the accumulation of sperm (white) and the oviduct contains oocytes that can be seen by transparency. Note a similar pigmentation of the oviduct (orange dots) in both *C. intestinalis* (E) and *C. roulei* (F). In *C. robusta* (G) and *C. edwardsi* (H), the spermiduct end is highly pigmented red and yellow respectively. The red coloration of the extrem tip of the vas deferens in *C. robusta* compare to the absence of coloration in *C. intestinalis* was already evocated by Sato et al. [22] .Images E to H are at the same scale (scale bar 500 µm in H).

(I-M) Oocytes. Images are at the same scale (scale bar 100 µm in L). *C. edwardsi* oocytes appear bigger than other.

(N-P) Larvae. Dorsal to the top, anterior to the left. Images are at the same scale (scale bar 200 µm in P).

References:

19 Brunetti, R. *et al.* Morphological evidence that the molecularly determined *Ciona intestinalis* type A and type B are different species: *Ciona robusta* and *Ciona intestinalis*. *J ZOOL SYST EVOL RES*, doi:10.1111/jzs.12101 (2015).

22 Sato, A., Satoh, N. & Bishop, J. D. D. Field identification of 'types' A and B of the ascidian *Ciona intestinalis* in a region of sympatry. *MAR BIOL* **159**, 1611-1619, doi:10.1007/s00227-012-1898-5 (2012).

28 Lahille, F. Faune ascidiologique de Banyuls-sur-Mer. *C.r. Soc. Hist. nat. Toulouse* **21**, 57-59. (1887).

52 Copello, M. *Ciona edwardsi* (Roule, 1886) espèce littorale de Méditerranée distinct de *Ciona intestinalis* Linne, 1767. *VIE MILIEU* **31**, 243-253 (1981).

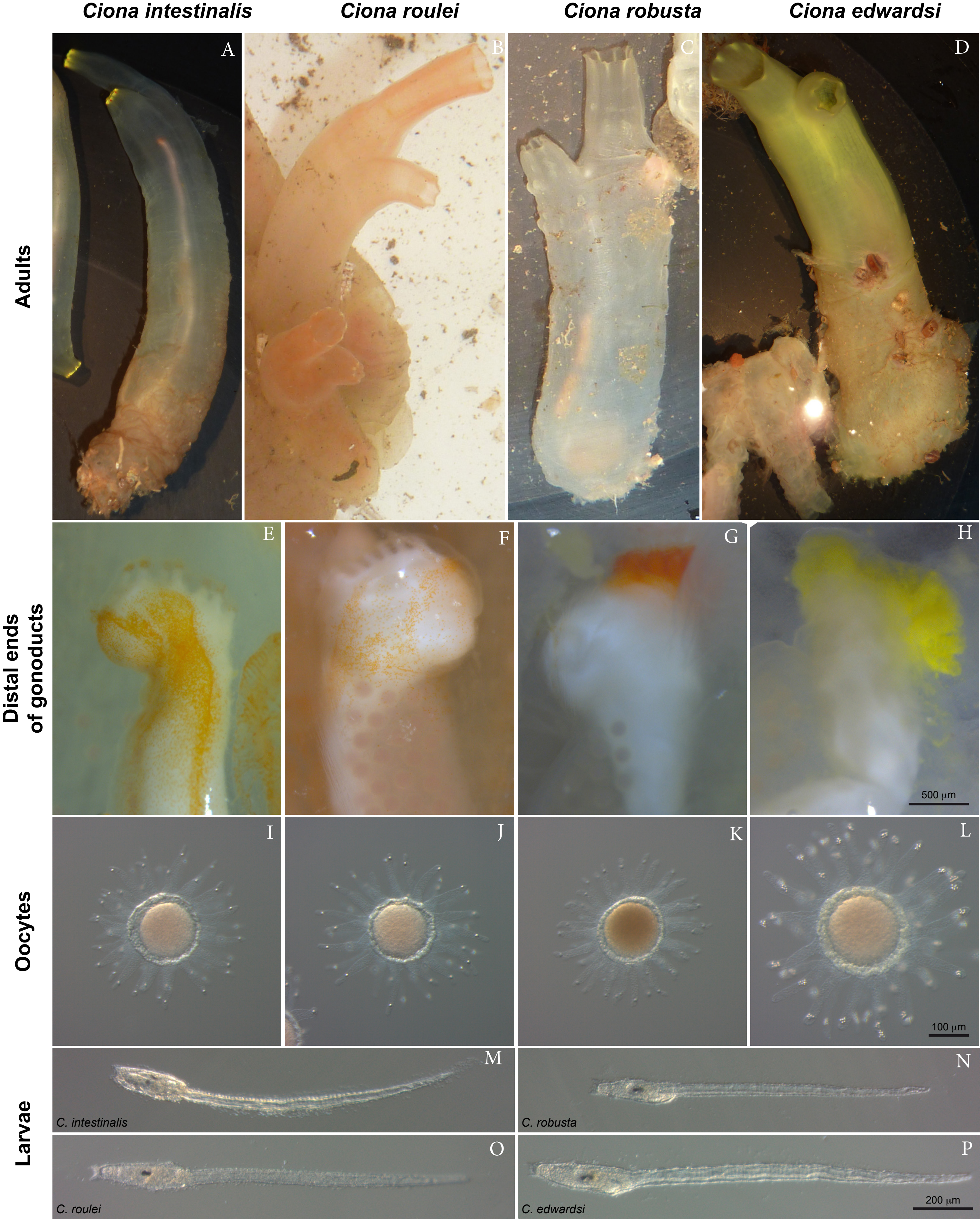
Ciona intestinalis
Ciona roulei
Ciona robusta
Ciona edwardsi
Adults
A
B
C
D
Distal ends of gonoducts
E
F
G
H
500 μm
Oocytes
I
J
K
L
100 μm
Larvae
M
N
C. intestinalis
C. robusta
O
P
C. roulei
C. edwardsi
200 μm
